# The No-touch Saphenous Vein Graft in Coronary Artery Bypass Surgery. Towards a New Standard?

**DOI:** 10.21470/1678-9741-2022-0955

**Published:** 2022

**Authors:** Walter J. Gomes, Ki-Bong Kim, Bruno Botelho Pinheiro, Domingos S. R. Souza

**Affiliations:** 1 Cardiovascular Surgery Discipline, Escola Paulista de Medicina and Hospital São Paulo, Universidade Federal de São Paulo, São Paulo, Brazil; 2 Cardiovascular Center, Myongji Hospital, Goyang-si, Gyeonggi-do, Republic of Korea; 3 Department of Cardiovascular Surgery, Hospital Israelita Albert Einstein, Goiânia, Goiás, Brazil; 4 Department of Cardiothoracic and Vascular Surgery, Faculty of Medicine and Health, Örebro University, Sweden

Saphenous vein grafts, along with the left internal thoracic artery graft, are the hallmark of coronary artery bypass grafting (CABG). Grounded on vast and landmark evidence, this technique remains a successful strategy for decades, with a significant advantage over medical or percutaneous intervention in treating advanced coronary artery disease (CAD), affording patients improved survival, reduction of myocardial infarction rates, and relief on angina symptoms.

However, the superior outcomes seen in coronary surgery are directly related to the graft’s ability to remain patent over the long term, averting spontaneous myocardium infarction, the main cause of death in CAD patients, besides relieving angina.

Offering the possibility of enhanced graft patency is consequently crucial for extending benefits and prolonging survival. Recently, tremendous efforts have been made in such a direction, pushing for wider utilization of arterial grafts. But the multiple arterial grafts (MAG) strategy has been met by coronary surgeons with skepticism and reluctance due to higher complications rate and resulting in low adherence practice^[^1^]^.

The introduction of the no-touch saphenous vein (NT-SV) graft, at first met with discredit and hesitancy, has been demonstrated by solid evidence (randomized controlled trials and meta-analyses) to offer superior patency than conventionally harvested vein graft over the long term, and comparable or even superior to MAGs^[^2^,^3^]^. The NT-SV technique is attractive because of the possibility of using a time-honored graft, familiar to all coronary surgeons, seemingly with a little change in harvesting technique. However, intrinsic and related complications with the NT-SV harvesting procedure have hindered broader acceptance, which must be acknowledged and adequately dealt with proper training. Thus, deviation from NT-SV basic concepts at harvesting and utilization may adversely affect long-term results, reinforcing the mandatory adherence to some core principles.

Bolstered by a new seminal study, CABG has sustained its ascent. The FAME-3 trial one-year results randomized three-vessel disease (3VD) patients to either CABG or fractional flow reserve-guided percutaneous coronary intervention (PCI) with current-generation zotarolimus-eluting stents. The most cutting-edge PCI technology was not effective in reducing major adverse cardiovascular and cerebrovascular events compared with CABG, reinforcing the advantages of CABG in 3VD patients. The findings are notable as the benefit of CABG was evident at the very early stage of the study, with just one-year follow-up, in contrast to previous studies where favorable results for CABG were only observed after longer-term follow-up^[^4^]^.

This Special Edition of the Brazilian Journal of Cardiovascular Surgery compiles the reports from the most experienced coronary surgeons worldwide working with this technique. While reinforcing the outstanding outcomes with the NT-SV grafts, it points to new strategies for dealing with complications of leg wound healing with innovative solutions, such as the minimally invasive or endoscopic harvesting techniques. Newly and delayed adopters will find instructive lessons for a soft start.

Finally, this Special Edition should serve as a call for collaborative work, strengthening the recent experience and further supporting this promising and effective technique.
